# Alcohol Use Disorder: The Role of Medication in Recovery

**DOI:** 10.35946/arcr.v41.1.07

**Published:** 2021-06-03

**Authors:** Barbara J. Mason, Charles J. Heyser

**Affiliations:** Pearson Center for Alcoholism and Addiction Research, Department of Molecular Medicine, Scripps Research Institute, La Jolla, California. Center for Human Development, University of California, San Diego, La Jolla, California

**Keywords:** disulfiram, acamprosate, naltrexone, gabapentin, medication-assisted treatments, alcohol use disorder, alcohol, drug therapy

## Abstract

The misuse of alcohol in the United States continues to take a large toll on society, resulting in the deaths of about 88,000 Americans per year. Moreover, it is estimated that nearly 14.6 million Americans currently meet diagnostic criteria for current alcohol use disorder (AUD). However, very few individuals receive treatment, with an even smaller portion receiving medications approved by the U.S. Food and Drug Administration (FDA) for the treatment of AUD, despite scientifically rigorous evidence showing the benefits of combining medication approved for treating AUD with evidence-based behavioral therapy. These benefits include higher rates of abstinence and less risk of relapse to heavy drinking, with associated improvements in medical and mental health and in quality of life. This review provides an overview of FDA-approved medications and “off-label” drugs for the treatment of AUD. The article emphasizes that AUD medical advice and prescription recommendations should come from professionals with training in the treatment of AUD and that treatment plans should consider medication in conjunction with evidence-based behavioral therapy. Finally, this review notes the limited number of medications available and the continued need for the development of new pharmacotherapies to optimize AUD recovery goals.

## INTRODUCTION

It is estimated that nearly 14.6 million Americans currently meet the diagnostic criteria for alcohol use disorder (AUD)^1^ included in the *Diagnostic and Statistical Manual of Mental Disorders*, 5th edition (*DSM-5*),^2^ and approximately 88,000 die from alcohol-related causes in the United States each year.^3^ An older term, “alcohol dependence,” is equivalent to the *DSM-5* criteria for AUD of moderate or greater severity.^4^ This is the stage of AUD severity for which pharmacotherapy is generally indicated. Effective intervention can decrease drinking and the likelihood of subsequent relapse, thereby significantly improving an individual’s health and reducing the negative consequences of AUD that are most likely to burden society.^5^

This paper provides an overview of the medications for AUD that are currently available for use by the recovery community, as well as a brief introduction to potential medications under development. Throughout, this review emphasizes that (1) all AUD medical advice and prescription recommendations should come from professionals (or in consultation with professionals) who have specific training in the treatment of AUD; (2) physical examination and laboratory testing are recommended before treatment is initiated and may help with subsequent monitoring of treatment response and adverse events; (3) medications are not “stand-alone” treatments for AUD, but rather are an element in a comprehensive treatment plan; (4) clinical trial data show drinking outcomes and recovery are significantly better when behavioral interventions are combined with AUD medication rather than given without AUD medication; and (5) there is a critical need for research on potential modifiers of response—including potential differences in drug metabolism due to sex hormones, race or ethnicity, and pharmacogenetic and pharmacometabolomic markers—to identify individuals most likely to respond or have significant side effects to specific AUD pharmacotherapies. The U.S. Food and Drug Administration (FDA) uses drinking outcomes of abstinence from alcohol and/or cessation of heavy drinking (males, five or more drinks per day; females, four or more drinks per day) in determining its approval of a candidate drug. Additionally, measures of improved medical and mental health and of quality of life are associated with these operational measures of recovery but often are not reported in the clinical trial literature given the relatively short duration of clinical trials (generally 6 months or less). Given that FDA approval is associated with drinking-specific outcomes and that these outcomes have been linked to improvement in measures of medical and mental health and quality of life, there is reason to believe that by alleviating problems associated with AUD, the use of AUD medications may bestow other positive contributions to recovery.^6^,^7^ The final section briefly reviews new pharmacological approaches and potential medications under development for the treatment of AUD.

## CURRENT FDA-APPROVED MEDICATIONS TO TREAT AUD

To date, the FDA has approved three medications for the treatment of AUD. These alcohol-specific pharmacotherapies are the oral alcohol-aversive drug disulfiram (Antabuse), introduced more than half a century ago in 1951; the opioid antagonist naltrexone, approved in 1994 as an oral formulation (Revia) and in 2006 as a long-acting injectable formulation (Vivitrol); and the oral centrally acting taurine analog, acamprosate (Campral), approved in 2004. In other countries, the European Medicines Agency approved the opioid antagonist nalmefene (Selincro) in 2013 for the treatment of alcohol dependence throughout the United Kingdom and European Union. Nalmefene is similar to naltrexone, but it binds more potently to a broader range of opioid receptor subtypes. The FDA-approved medications act via widely different mechanisms but share some key features relevant to recovery and highlight the complex nature of AUD. More specifically, these medications are aimed at restoring normal functioning in alcohol-altered neurophysiological processes or act to blunt or punish the reinforcing properties of alcohol.

Treating AUD with a prescribed drug can appear counterintuitive or concerning to those aspiring to a drug-free recovery. Therefore, such overarching concerns must be addressed before delving into the details of a specific medication. All drugs (prescribed, herbal, and over-the-counter) have a potential for harm. FDA has evaluated the drugs approved to treat AUD and found the safety profile to be acceptable, particularly given the potentially lethal harms of ineffectively treated AUD of moderate or greater severity. None of these prescribed medications are mood-altering, habit-forming, or addictive. They do not produce euphoria or other subjective experiences associated with misuse potential, nor do they have “street value” as do illicit drugs. None are “substitution” drugs for alcohol, as is methadone for heroin. Tolerance, or a need to increase the dose, does not develop with continued use, nor does rebound craving or drinking occur when medication is discontinued.

All AUD medical advice and prescription recommendations should come from professionals (or in consultation with professionals) who have specific training in the treatment of AUD. This training is critical because the efficacy of drug treatment may be influenced by an individual’s unique characteristics, including comorbid conditions, severity and complexity of AUD, state of sobriety at the onset of treatment, medication adherence, any side effects, and motivation to recover from AUD. Treatment outcomes in a large acamprosate trial were significantly better in individuals motivated to a treatment goal of abstinence.^8^ Therefore, a detailed understanding of these factors and available treatment options, obtained in partnership and communication with the individual, may optimize treatment selection and recovery. In addition, and perhaps more important, the time course for recovery is quite variable and subject to myriad environmental changes. Therefore, a trained professional is in the best position to respond to these changes in real time and adjust treatment accordingly.

People in recovery from AUD may need to take medications for other medical or psychiatric disorders, in conjunction with medication for AUD. Physician members of Alcoholics Anonymous (AA) have developed a thoughtful guide to the appropriate use of such non-AUD medications, with the aim of minimizing risk of their misuse and undermining recovery. Both treatment providers and persons in recovery can refer to and access the guide online ( https://www.aa.org/pages/en_US/aa-member-medications-and-other-drugs). AA does not offer medical advice, but strongly recommends seeking out physicians who are experienced in the treatment of AUD. Persons in recovery are urged to communicate openly with their prescribing doctor if they skip doses or take extra medication, have a desire to take more medication, or experience side effects that make them feel worse, as well as to be sensitive to changes in their own behavior and mood when starting a new medication or when a dose is changed. Such reactions could signal an increased risk of drug misuse or relapse. AA stipulates that its members do not “play doctor”; all medical advice and prescriptions should come from a qualified provider.

## EFFICACY CRITERIA FOR MEDICATIONS TO TREAT AUD

Comprehensive meta-analyses of randomized controlled trials of FDA-approved medications to treat AUD have shown a significant benefit on rates of abstinence and/or cessation of heavy drinking in studies that were typically 6 months in duration (see Table 1). It is critical to appreciate that those clinical trials included either the nonpharmacological treatment routinely provided for AUD in a given setting or protocol-specific behavioral treatments for all participants. Therefore, the medication (plus behavioral treatment) demonstrated a significant benefit over placebo (plus behavioral treatment) on drinking outcomes.

These rigorous, evidence-based findings have two important implications:

1. Medications are not “stand-alone” treatments for AUD, but rather an element in a comprehensive treatment plan that includes behavioral therapy.
2. Drinking outcomes are significantly better when behavioral interventions are combined with AUD medication than when they are given without AUD medication.

Clinical trials of AUD medications typically incorporate a derivation of motivation enhancement or cognitive-behavioral treatment manuals developed for Project MATCH ( https://pubs.niaaa.nih.gov/publications/projectmatch/matchintro.htm ); the manual used in the multicenter U.S. acamprosate study is available at http://www.pearsoncenter.org/therapistmanual.

Given the incremental gains in recovery found when AUD medications are used in combination with behavioral treatment, recovery strategies should consider medications as an option in the treatment plan for AUD. For individuals with AUD, recovery historically has been viewed as a lifestyle of voluntary abstinence from alcohol and nonprescribed drugs.^9^ In addition to complete abstinence, FDA has identified “no heavy drinking” as a clinically relevant outcome for assessing a drug’s efficacy for AUD, given the relationship between alcohol-related harms and heavy drinking. Chronic heavy drinking is defined in women as routinely drinking more than three drinks per day or more than seven drinks per week, and in men as routinely drinking more than four drinks per day or more than 14 drinks per week.^10^ These two FDA-recognized outcomes can be reported as the percentage of individuals having no drinks or no heavy drinking days over the course of treatment, which is typically 6 months in duration (see Table 1).

A third potential regulatory outcome for approval of a drug for treatment of AUD has recently been proposed. The proposed outcome involves a reduction of one or two in the World Health Organization (WHO) risk levels of alcohol use (measured in grams of alcohol consumed per day).^11^ The European Medicines Agency used this outcome in its evaluation of nalmefene for the treatment of AUD.^12^ Of note, unlike other oral AUD medications, nalmefene is not taken daily, but rather 2 hours prior to an anticipated heavy drinking situation. The 6-month duration of the majority of clinical trials for AUD may be too brief and the sample sizes too small to measure alcohol-related harms, such as driving under the influence or impaired quality of life. However, secondary analyses of larger data sets have shown that a reduction in WHO risk drinking levels is associated with significantly fewer alcohol-related consequences (e.g., less anxiety and depression, lower blood pressure and liver enzyme levels, improved quality of life).^6^,^7^ Taken together, these findings suggest that the significant benefits of FDA-approved medications on reduced alcohol consumption also may have wide-ranging emotional and physical health benefits for individuals with AUD.

## INTEGRATING MEDICATION INTO AN AUD TREATMENT PLAN

Given the scope of benefits associated with pharmacotherapy combined with evidence-based behavioral treatment for AUD, it is perplexing that a nationwide pharmacy survey suggests that fewer than 9% of eligible individuals have ever been provided with a prescription for a medication to treat AUD; psychiatrists provided the majority of these prescriptions.^13^ Recent large-scale meta-analyses have reported that either acamprosate or naltrexone combined with counseling has superior efficacy for increasing rates of abstinence or of no heavy drinking relative to counseling administered in conjunction with placebo.^14^,^15^ Recognizing the incremental gain typically achieved when medication is incorporated into the treatment plan, the American Psychiatric Association (APA) recently developed a practice guideline for the pharmacological treatment of individuals with AUD.^16^ This guideline suggests that acamprosate or naltrexone be used in individuals with moderate to severe AUD who wish to cut down or quit drinking, who prefer medication or who have not responded to nonpharmacological treatments, and who have no contraindications to the use of these medications. APA further suggests that disulfiram should not be selected as an initial treatment for AUD, given the physiological consequences of drinking in combination with this medication. In addition, this guideline recommends that antidepressant medications should not be used for the treatment of AUD, unless there is a comorbid disorder for which these treatments are indicated.^16^ Furthermore, the medications approved to treat AUD are not treatments for alcohol withdrawal and should be initiated only following detoxification and/or after abstinence has been established. Acute withdrawal involves primarily symptoms of autonomic hyperactivity that may last up to 5 days, and although most cases (85%) do not require medication, severe alcohol withdrawal can be life-threatening if untreated.^17^ Benzodiazepines are a standard treatment for clinically significant acute alcohol withdrawal symptoms, with the understanding that they are not an accepted treatment of AUD per se because of misuse potential.^16^

In its first report on alcohol, drugs, and health, the Office of the Surgeon General proposes a chronic care management approach to AUD that includes evidence-based behavioral and pharmacological treatments; social support services; and clinical monitoring of adverse events, medication adherence, and symptoms of relapse at every follow-up visit.^18^ The report notes the importance of working collaboratively with the individual and their social support system; communicating the risks and benefits of each treatment option relative to the individual’s recovery goals, drug costs, and dosing schedule; and ensuring that the individual comprehends this information. This again serves to highlight the importance of specific training in the treatment of AUD, given the need to explain complex information using clearly understood language. A written information sheet providing details about the prescribed medication can be taken home by the individual for future reference. It is recommended that the provider contact the individual a few days after an AUD medication is prescribed to address any concerns, to assess medication adherence and side effects, and to facilitate successful medication initiation.

## SAFETY AND SIDE EFFECTS OF AUD MEDICATIONS

The well-being and safety of the individual is always the highest concern. Each AUD medication has a label or package insert that contains FDA-approved statements about the drug’s indication (or purpose), dosing, side effects, and any warnings or contraindications. The label can be accessed by typing “[drug name] label” in an online search engine. Safety is optimized by heeding the recommended dose and the cautions and contraindications on the drug label. Ideally, the provider would have access to a complete and detailed medical history of the individual to optimize safety. Physical examination and laboratory testing are recommended before treatment is initiated and may help with subsequent monitoring of treatment response and adverse events. These lab tests could include alcohol breath/blood concentration, alcohol glucuronide testing, urine drug screen, liver function tests (i.e., gamma glutamyl transferase [GGT], alanine transaminase, aspartate transaminase), complete blood count, testing for vitamin deficiencies, renal function tests (standard panel for urea [blood urea nitrogen], electrolytes, and serum creatinine), and a pregnancy test for women of childbearing potential. Furthermore, measures of hepatic function and creatinine clearance may be critical in determining the choice of drug treatment. For example, baseline liver function tests may detect clinically significant hepatic impairment that would mitigate against treatment with disulfiram and naltrexone as well as severe impairment in creatinine clearance that would contraindicate the choice of acamprosate. A baseline urine drug screen may also be useful, as it may provide information about otherwise undisclosed drug use, including opioid use, which would rule out naltrexone treatment of AUD.

Individuals also should be assessed for any comorbid disorders, including depression and other drug use disorders. Comorbid conditions may significantly influence AUD outcome if left untreated. Risk of suicide may be elevated in individuals with AUD, and it is recommended that the individual be screened and monitored for suicidality at baseline and throughout treatment to identify increased suicide risk that requires further intervention.

As with all medications, the FDA-approved pharmacotherapies for the treatment of AUD have common side effects (e.g., dizziness, nausea, diarrhea). Usually mild and associated with treatment initiation, these side effects resolve quickly. Individuals should be advised to avoid driving a car or operating heavy machinery until they are reasonably certain that the drug does not affect their ability to engage in such activities. Individuals should be given emergency phone numbers and instructed to call immediately if suicide ideation or depression develops, or if symptoms of acute hepatitis or liver failure emerge (in the case of naltrexone and disulfiram). As a precaution, it is highly recommended that individuals carry a card in their wallet listing all current medications in the event of a medical emergency. For example, anesthesia and pain management may need to be adjusted in individuals taking naltrexone. Furthermore, the presenting medical emergency may be the result of an interaction between alcohol and disulfiram.

Medication nonadherence will negatively impact treatment outcomes. Individuals can be instructed to bring the container for their oral medication to follow-up visits to be assessed for unused drug. Noncompliance can result from adverse side effects, inconvenience, the perception that the drug is no longer needed (i.e., “I feel fine”), and/or a return to drinking. It is therefore critical to understand the reason(s) for treatment noncompliance. First, treatment providers need to determine if adverse events (e.g., medication side effects) are undermining medication adherence, and intervene accordingly. In terms of convenience, long-acting injectable naltrexone was developed to offset the adherence problems noted with daily oral naltrexone dosing. Given that acamprosate has a dosing schedule of three times daily, it is recommended that patients keep their medication in a weekly pill organizer with day and time indicated for each dose. Patients are also advised to link commonly missed doses with an activity of daily living such as eating meals or brushing teeth as a reminder to take their medication at that time. Monitoring medication compliance is paramount to successful treatment outcomes.

## MEDICATION INITIATION AND DURATION

The early days of abstinence are a period of heightened vulnerability for relapse and a critical time for healing neural processes associated with negative affect and impaired executive function.^19^ Medications for AUD can have the greatest impact on reducing relapse risk when initiated immediately after a 4- to 7-day detoxification period.^15^,^20^

The patient’s pattern of alcohol misuse should be established as a baseline, preferably using quantitative self-report and biochemical measures, against which treatment effects can be tracked. In addition, harmful effects of alcohol on the individual’s health, functioning, and legal status should be documented and incorporated into a personalized treatment plan.

There is little scientific evidence to guide the optimal duration of pharmacological treatments of AUD. Decisions about treatment duration should reflect the individual’s history of relapse, the severity of AUD at baseline, and the individual’s clinical response and side effects to the medication. This should be discussed with the individual if they express a desire to discontinue treatment before a stable recovery has been achieved.

In situations where there is no response to treatment, the provider may consider switching to an alternative AUD medication. This decision is more difficult in situations where a partial response is observed. For example, an individual may have reduced their drinking by half from baseline, but continues to have episodes of heavy drinking. In these situations, the provider may consider the use of combined treatments on a case-by-case basis. Some data lend support to the safety of acamprosate combined with naltrexone or disulfiram,^21^–^23^ but efficacy data are insufficient to support a general recommendation for combined use as a first-line treatment approach to AUD.^16^

## FDA-APPROVED MEDICATIONS FOR AUD

### Disulfiram

**Figure 1:**
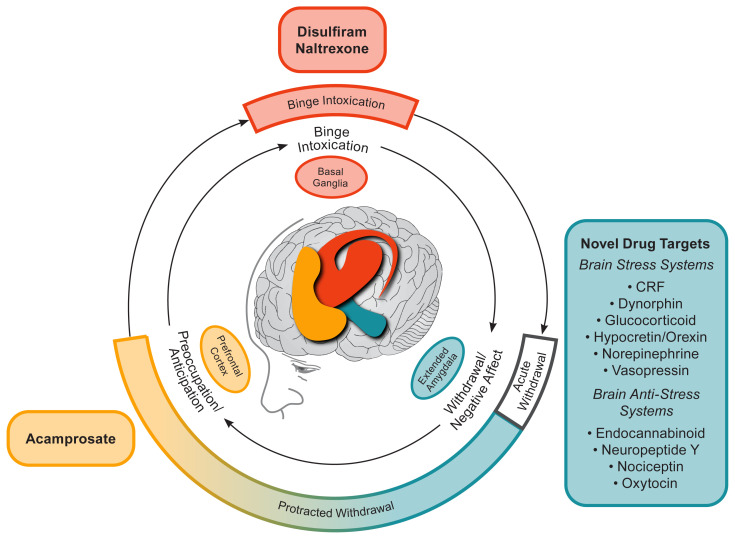
Conceptual framework for the effects of various medications on the three major stages of the alcohol addiction cycle and the clinical stages of alcohol use disorder (AUD).The outer ring relates to clinical stages of AUD.The inner ring relates to three stages of the addiction cycle. Acute withdrawal relates to physiological and emotional effects that are opposite to those of alcohol and includes activation of the extended amygdala brain stress systems. Acute withdrawal is a time-limited process (up to only 5 days in duration).Protracted withdrawal is characterized by continued hyperactivation of the brain stress systems. The overexpression of brain stress neuropeptides is hypothesized to mediate the anxiety, dysphoria, irritability, and sleep disturbances of post-acute (i.e., protracted) withdrawal that may persist for an indefinite duration. Protracted withdrawal/negative affect helps drive craving in the preoccupation/anticipation stage, for which acamprosate is the only available treatment. *Note*: CRF, corticotropin-releasing factor. Adapted by permission from Springer Nature: Nature Neuropsychopharmacology, 35(1):217–38, Neurocircuitry of addiction, George F. Koob and Nora D. Volkow, 2010.^31^

In 1951 disulfiram (Antabuse; now in generic formulations) was the first drug approved for the treatment of AUD by the FDA. Pharmacologically, disulfiram inhibits the enzyme aldehyde dehydrogenase. Even small amounts of alcohol can cause acetaldehyde to quickly accumulate, resulting in a rapid onset of flushing, nausea, and vomiting. The resulting acute physical distress serves to reduce drinking and break the cycle of binge intoxication (see Figure 1). In severe reactions, there is the possibility of multiple cardiac and respiratory symptoms that could result in death. The intensity of the interaction varies across individuals but is generally proportional to the amounts of disulfiram and alcohol ingested and can last from 30 to 60 minutes to several hours, or as long as there is alcohol in the blood. Individuals should be instructed to abstain from alcohol for at least 12 hours before taking disulfiram and be advised that reactions with alcohol can occur up to 14 days after discontinuing disulfiram.

The therapeutic action of disulfiram is punitive, resulting in acute physical distress when taken with alcohol. Therefore, it should never be given to an individual in a state of alcohol intoxication or without their full knowledge. When taken as prescribed, disulfiram is typically well tolerated,^24^ but more serious adverse events were found with disulfiram than with comparison treatments.^25^ The psychological threat (fear) of the interaction between disulfiram and alcohol may be the primary mechanism of disulfiram’s deterrent effect, as opposed to the drug’s pharmacodynamic properties.^25^ Therefore, consideration of disulfiram may be warranted only in individuals who have a clear goal of complete abstinence, are capable of understanding the risks of an interaction between alcohol and disulfiram, have not responded to acamprosate and naltrexone, and have no medical contraindications.^16^ Given the drug’s potential for hepatotoxicity, it is recommended that individuals taking disulfiram have bilirubin and liver function tests at baseline and 2 weeks, once a month for the next 6 months, and every 3 months thereafter. Medication nonadherence is a common problem with disulfiram,^26^ and outcomes are optimized with supervised administration.^27^

### Naltrexone

Naltrexone is a pure opioid receptor antagonist that the FDA approved first for opioid dependence (in 1984), and later for alcohol dependence (as an oral medication in 1994 and as a long-acting injectable in 2006). The therapeutic action of opioid receptor antagonism is to blunt the rewarding effects of alcohol. In our conceptual model (shown in Figure 1 ), blunting the rewarding effects of alcohol can reduce drinking and break the cycle of binge intoxication. Although side effects are generally mild (initial nausea, vomiting, and dizziness), a recent meta-analysis found a higher risk for discontinuation due to adverse events with naltrexone relative to placebo.^14^ This meta-analysis, which included the results of 53 randomized controlled trials (involving 9,140 patients) of oral naltrexone (50 mg/d) for the treatment of AUD, showed that naltrexone significantly decreased the likelihood of a return to heavy drinking and, to a lesser extent, a return to any drinking.^14^ This replicated the results from an earlier meta-analysis that reported a decreased risk of a return to heavy drinking and that also assessed moderators of naltrexone treatment response.^15^ Maisel et al. (2013) found that 4 days of abstinence prior to beginning treatment significantly improved naltrexone treatment response and that having treatment goals other than abstinence was associated with a larger effect size on reducing heavy drinking than having the goal of complete abstinence.

Naltrexone, like disulfiram, is pharmacologically effective primarily while present in the system, but induces no long-term changes in the brain.

This is important in understanding the duration of treatment effects of naltrexone and disulfiram. For example, follow-up studies of patients in two 3-month naltrexone studies showed that treatment effectswerenolongersignificantrelativeto placebo by 1 to 3 months posttreatment.^28^,^29^ Pairing naltrexone with a form of cognitive behavior therapy focused on relapse prevention coping skills, therefore, may offer an optimal treatment strategy.^29^

Regarding route of administration, there have been no head-to-head comparisons of the efficacy of oral versus injectable naltrexone to date. A meta-analysis of drinking outcomes from 1,926 participants in two trials of different formulations ofinjectablenaltrexonefoundnosignificanteffects of treatment on return to any drinking or to heavy drinking,butdidfindareductioninthenumber of heavy drinking days. The trial conducted in support of FDA approval found a similar effect of naltrexone (Vivitrol) 380 mg per injection, but only in men and only in those with 7 days of abstinence prior to randomization.^20^

Any form of naltrexone treatment for AUD is contraindicated in individuals who have current physiologic dependence on opioids, who are in opioid withdrawal, who have used prescribed or illicit forms of opioids within the past 7 to 10 days, or who have a urine drug screen positive for opioids. This avoids unintended precipitation of opioid withdrawal through administration of an opioid antagonist. Of note, naltrexone can cause hepatocellular injury when used in higher than recommended doses and is contraindicated in individuals with acute hepatitis or liver failure.

### Acamprosate

Acamprosate was developed in France in the 1980s and approved by FDA for the maintenance of abstinence in detoxified patients with alcohol dependence in 2004. The pharmacological action of acamprosate is complex. The chemical structure is similar to that of the endogenous amino acid homotaurine, which is a structural analog of the amino acid neurotransmitter gamma-aminobutyric acid (GABA) and the amino acid neuromodulator taurine. Repeated cycles of heavy drinking and withdrawal have been shown to dysregulate the balance between neuronal excitation (e.g., glutamatergic) and inhibition (e.g., GABAergic).^30^ It has been hypothesized that this glutamatergic hyperactivity is associated with alcohol craving and the preoccupation/anticipation phase of protracted withdrawal— an effect that is ameliorated by acamprosate (see Figure 1).^31^ Therefore, it suggested that the pharmacotherapeutic action of acamprosate in AUD works by restoring homeostasis in *N*-methyl-D-aspartate (NMDA)–mediated glutamatergic neurotransmission.^32^,^33^ Acamprosate requires approximately 1 week to reach steady-state levels in the nervous system, and its effects on drinking behavior have been shown to persist in studies of up to 1 year after the treatment is completed, consistent with its role in restoring persisting homeostasis in brain glutamatergic activity.^33^

A meta-analyses of 27 randomized controlled trials of acamprosate (typically 6 to 12 months in duration) found that acamprosate was significantly more likely than placebo treatment to prevent a return to any drinking.^14^ This finding replicates the results from an earlier meta-analysis that found a significantly higher rate of complete abstinence associated with acamprosate.^15^ Detoxification or required abstinence prior to acamprosate administration was associated with increased efficacy.^15^ A separate meta-analysis using individual records from more than 6,000 participants in 22 acamprosate studies found the medication to have a significant gain in the rate of complete abstinence and no heavy drinking over the study duration,^34^ with no differences in the rate of discontinuation due to adverse events or severity or type of adverse event. Acamprosate was also associated with significantly higher rates of treatment completion and medication compliance than placebo. Posttreatment follow-up studies have shown the effects of acamprosate to be sustained for periods of up to 1 year after the last dose.^33^ Acamprosate also has been reported to reverse alcohol-related insomnia and changes in sleep architecture.^35^,^36^ This added benefit may improve treatment outcomes in individuals with comorbid psychiatric disorders characterized by sleep disturbance, such as post-traumatic stress disorder, anxiety, and depressive disorders.

Acamprosate is not metabolized by the liver and is not associated with hepatotoxicity. moreover, acamprosate does not interact with medications commonly prescribed for individuals with AUD, including disulfiram, antidepressants, anxiolytics, or hypnotics. pharmacokinetic studies found that coadministration with naltrexone increased the rate and extent of acamprosate absorption without compromising its tolerability.^22^,^23^ As noted previously, acamprosate is taken three times a day, due to low bioavailability. This dosing schedule may be supported by placing a 1-week supply of medication in a commercially available pill organizer with day and time indicated for each dose. Acamprosate is well tolerated with minimal side effects (e.g., mild to moderate diarrhea, typically at the start of treatment). The results of a meta-analysis found acamprosate to have no increase in the risk of withdrawal from treatment due to adverse events compared with placebo.^14^

## “OFF-LABEL” MEDICATIONS TO TREAT AUD

Given that existing pharmacotherapies are underutilized and limited in scope, there is a continued need for the development of new medications to treat AUD safely and effectively. One avenue to discovery involves the repurposing of existing medications. This is the most expeditious route given that these drugs have FDA approval for use as treatments in other medical conditions and known safety profiles. However, once a drug is in generic formulations, there is little financial incentive for a pharmaceutical company to incur the cost of the additional research required for FDA approval of AUD as a new indication. Thus, the use of such drugs to treat AUD is considered “off label.” Two generic drugs, topiramate and gabapentin (both originally developed as antiepileptic medications), have shown therapeutic potential for AUD and have been included in APA’s practice guideline.^16^ The guideline recommends the use of topiramate or gabapentin in individuals who have a goal of decreasing or quitting drinking and who are intolerant to or have not responded to acamprosate and naltrexone.^16^ Co-occurring disorders, concomitant medications, side effect profiles, and contraindications for use are additional factors that may guide the selection of topiramate or gabapentin.

### Topiramate

Topiramate (Topamax and generics) is currently approved by the FDA for the treatment of epilepsy and for the prophylaxis of migraine, and has been extensively studied for the treatment of AUD. A meta-analysis of randomized controlled trials of 3 months duration and target doses of 200 to 300 mg/d in outpatients with AUD found topiramate to be associated with fewer drinking days, fewer heavy drinking days, and fewer drinks per drinking day, compared with placebo.^14^ Although promising, topiramate has a number of warnings and precautions. Safety monitoring recommends baseline and periodic measures of serum bicarbonate to detect treatment-emergent metabolic acidosis; baseline tests of renal function, as creatinine clearance of less than 70 mL/min requires a dose adjustment to half the starting and maintenance dose; and baseline tests of hepatic function, as topiramate plasma concentration is increased in hepatic impairment. In addition, it has been reported that individuals with AUD who were treated with topiramate had a higher risk of cognitive dysfunction, paresthesia, and taste abnormalities than did individuals treated with placebo. The cognitive dysfunction—including confusion; psychomotor slowing; attention, concentration, and memory impairment; and speech or language problems—was commonly associated with treatment discontinuation.^37^ Individuals should be gradually withdrawn from topiramate to minimize the potential for seizures. An individual’s current medications should be reviewed prior to considering topiramate, which interacts pharmacokinetically with some antiepileptic drugs, central nervous system depressants, oral contraceptives, metformin, lithium, and carbonic anhydrase inhibitors.

### Gabapentin

Gabapentin (Neurontin and generics) is used “off label” for the treatment of AUD and is included in APA’s practice guideline.^16^ It is a synthetic GABA analog approved by FDA for the treatment of epilepsy and postherpetic neuralgia.^38^ The authors hypothesize that gabapentin acts in AUD to break the cycle of negative affect given its effects on mood and sleep and on electrophysiological results showing that it acts like a corticotropin-releasing factor (CRF) receptor antagonist in the central nucleus of the amygdala (CeA)^38^ (see Figure 1). A recent review found the efficacy of gabapentin for treatment of AUD supported by five of six single-site treatment studies reporting drinking outcomes.^39^ The efficacy of gabapentin has been reported to be dose dependent. More specifically, a 12-week trial of 0, 900, and 1,800 mg/d of gabapentin showed significant linear dose effects on rates of abstinence and absence of heavy drinking; number of drinks per week; number of drinking days per week; GGT; and standardized measures of craving, negative affect, and insomnia,^40^ with the 1,800 mg/d dose associated with greatest efficacy. Similar to acamprosate, six of eight AUD studies reported a significant beneficial effect of gabapentin on alcohol-related sleep disturbance.^39^ Moreover, gabapentin-related decreases in negative affect have been reported.^39^ These clinical findings are consistent with basic research suggesting gabapentin may support recovery by restoring homeostasis (a stable equilibrium) in brain stress systems that become dysregulated in the protracted withdrawal/negative affect phase of AUD.^38^ Research suggesting that gabapentin may be most effective in individuals with acute alcohol withdrawal symptoms was challenged because individuals with clinically significant acute alcohol withdrawal were systematically excluded from participation in this research.^41^ Gabapentin should not be considered a standalone treatment for severe acute alcohol withdrawal because of its ineffectiveness in suppressing seizures related to alcohol withdrawal.^39^ The APA practice guideline recommends the use of gabapentin for the treatment of AUD, not alcohol withdrawal.^16^ Note that relative to other AUD medications, gabapentin shows unique evidence for treating the mood and sleep disturbance of the protracted withdrawal phase.

There are no contraindications to gabapentin, other than known hypersensitivity to the medication. Gabapentin is not metabolized in the liver and is eliminated from systemic circulation by renal excretion as unchanged drug. As such, a baseline test of creatinine clearance is indicated, with dose adjustments indicated in individuals with reduced renal function (creatinine clearance < 60 mL/min). Alcohol was not found to interact meaningfully with gabapentin in a pharmacokinetic/pharmacodynamic (PK/PD) study.^42^ The lack of appreciable hepatic metabolism is a PK advantage of gabapentin, as chronic heavy drinking is often associated with liver injury. There were no reported safety concerns among the 655 individuals with AUD treated with gabapentin in clinical studies (≤ 1,800 mg/d), and any adverse events tended to be mild to moderate and to not differ from placebo.^39^ These common adverse events included headache, insomnia, fatigue, muscle aches, and various gastrointestinal complaints at equivalent rates in both gabapentin- and placebo-treated outpatients with AUD. Taken together with patient experience for approved pain and epilepsy indications, gabapentin is considered to have a good safety and tolerability profile. As with any centrally active drug, individuals should be advised not to drive motor vehicles or operate heavy machinery until they have ascertained that the drug does not affect their performance.

Antiepileptic drugs, including gabapentin and topiramate, have been shown to increase the risk of suicidal thoughts or behavior in about one in 500 patients, irrespective of disorder. Further, abrupt withdrawal from gabapentin and topiramate can increase the risk of precipitated seizures and status epilepticus, and drug dose should be tapered gradually when discontinuing treatment. Reports of misuse of gabapentinoids, such as gabapentin and pregabalin, are increasingly documented in high-risk populations, notably among those who misuse opioids and prescription drugs. Gabapentin is not a controlled or scheduled substance. There was no evidence of tolerance to gabapentin dose or rebound with titration off drug, nor evidence of misuse potential, in studies of individuals with AUD. However, patients undergoing opioid withdrawal, those who misuse prescriptions recreationally, and prison populations may be at increased risk to misuse gabapentin, with self-administered doses often far exceeding the therapeutic range.^43^,^44^ Hence, patients with risk histories should be monitored for potential gabapentinoid misuse or diversion.

### Baclofen

Baclofen is a selective gamma-aminobutyric acid-B (GABA-B) receptor agonist; see de Beaurepaire et al., 2019, for review.^45^ Baclofen has been used to treat muscle spasticity, secondary to neurological conditions. It has been hypothesized that the pharmacotherapeutic action of baclofen in AUD may be to suppress the ventral tegmental area (VTA) dopamine system and blunt reinforcement, serving to reduce drinking and thereby breaking the cycle of binge intoxication (see Figure 1). Initial reports were positive in 39 male participants with AUD, showing that treatment with baclofen 30 mg/d increased the percentage of individuals who achieved and maintained abstinence as well as the number of abstinent days, and decreased the number of drinks per drinking day as well as anxiety levels.^46^ However, these results have not been consistently observed in subsequent studies.^45^ In addition, the use of baclofen remains controversial, in part because of uncertainty regarding dosing and efficacy, along with concerns about safety. Individuals should be told to avoid drinking while taking the drug as the sedative properties of both drugs may potentiate each other. Individuals should not drive motor vehicles or operate heavy machinery until they have ascertained that the drug does not affect their performance. Individuals also should be advised of the risk of overdose. Side effects range in severity, from nonsevere to more dangerous types, including seizures, respiratory depression with sleep apnea and potentially coma (in case of intoxication), severe mood disorders (mania or depression, with the risk of suicide), and mental confusion or delirium. Baclofen is mostly (~ 80%) eliminated from systemic circulation by renal excretion as unchanged drug. Therefore, baseline and repeated tests of renal function are recommended given that renal problems can lead to an accumulation of baclofen, which may result in mental confusion. Baclofen treatment should start and end slowly as there is a withdrawal syndrome associated with abrupt cessation of treatment; withdrawal symptoms may include confusion, agitation, seizures, and delirium and may be confused with alcohol withdrawal.^47^ More research is needed to clarify the potential efficacy and safety of baclofen in AUD.

## SEX DIFFERENCES IN AUD AND RESPONSE TO AUD PHARMACOTHERAPIES

To date, very few publications have examined sex differences in pharmacotherapies for AUD. This is surprising given that 5.6 million American women (~4%) met criteria for AUD in a recent survey by the Substance Abuse and Mental Health Services Administration.^48^ Furthermore, it has been reported that women generally experience liver damage and other health problems after consuming less alcohol than men.^49^,^50^ For example, among women, chronic consumption of more than two drinks per day is associated with increased risk of mortality, breast cancer, hypertension, stroke, and reproductive problems;^49^ and binge drinking (e.g., consuming four or more drinks in a row) may incur increased risk of accident, rape, assault, and unprotected sex.^51^ Given the significant disease burden of AUD in women, early intervention and effective treatment options are imperative.

There is a clear need for women to be represented in clinical trials of AUD, because sex may be associated with differential drug efficacy. The majority of clinical trials of disulfiram have been conducted primarily in men; women comprised less than 10% of all patients included in a recent meta-analysis.^25^ A clear example of sex differences was reported in a pivotal multicenter trial for AUD where long-acting injectable naltrexone (Vivitrol) showed efficacy in men but not in women.^20^ The reason for the sex difference in Vivitrol efficacy is not understood, as the pharmacokinetics of the drug do not differ between men and women. Additionally, oral naltrexone did not differ from placebo in the only trial exclusively studying women.^52^

Conversely, no sex differences were found in a sex-specific meta-analysis of individual records obtained from 1,317 women and 4,794 men who participated in 22 acamprosate clinical trials.^34^ A significant effect of acamprosate relative to placebo on rates of abstinence and absence of heavy drinking was found in both men and women. The side effect and tolerability profile of acamprosate was comparable to that of placebo and did not differ between women and men. Acamprosate was associated with significantly higher rates of treatment completion and medication compliance than placebo among both women and men.

Systematic evaluation of potential differences in drug metabolism due to race, ethnicity, or sex hormones, and of consequent effects on drug efficacy or safety, is essential for all medications to treat AUD, and clinical trials require adequate representation of women and individuals from diverse racial and ethnic backgrounds. An additional concern is that the prevalence of AUD is highest among women in the prime childbearing years (ages 18 to 29), with associated risk of fetal alcohol spectrum disorders.^53^ Women with childbearing potential who do not use a reliable method of birth control or who are pregnant or lactating must be excluded from medication trials to avoid exposing the fetus or newborn to medication. There are no adequate and well-controlled studies of pharmacotherapies for AUD in pregnant women. Therefore, it is recommended that these medications not be used during pregnancy.

## PHARMACOGENETIC AND PHARMACOMETABOLOMIC PREDICTORS OF RESPONSE

Pharmacogenetic and pharmacometabolomic predictors have the potential to inform clinical care by identifying individuals likely to respond to or have significant side effects to a specific medication, thereby personalizing AUD treatment. For example, a number of pharmacogenetic studies have focused on the moderating effects of a variant in the mu-opioid receptor gene OPRM1 on response to naltrexone. However, a comprehensive review of the literature concluded that inconsistent findings across studies and a lack of translation of findings from human laboratory studies to clinical trials do not yet support this application of pharmacogenetics in AUD clinical practice.^54^

Recent studies using pharmacometabolomics offer insights into optimizing acamprosate treatment. For example, elevated baseline serum glutamate was found to be a biomarker of response to acamprosate in alcohol-dependent patients,^55^ with responders showing significantly higher baseline serum glutamate levels. Interestingly, this study reported that serum glutamate levels of responders were normalized after acamprosate treatment, whereas there was no significant glutamate change in nonresponders; this provides further support for the hypothesis that acamprosate works to restore homeostasis in the brain glutamate system. By developing such predictors, it may be possible to improve patient treatment matching and the overall success rate of acamprosate—and, to that end, any pharmacotherapy used in the treatment of AUD.

## CONCLUDING REMARKS AND FUTURE DIRECTIONS

The recent surge in understanding of the neurocircuitry and neuropharmacological mechanisms that are involved in AUD have provided abundant targets for future medication development for treating AUD.^31^ However, most previous work on medications has focused on blocking the rewarding effects of drugs in the binge intoxication stage of the AUD cycle. A clear role for drug targets in the protracted withdrawal phase is indicated by persisting negative emotional states that drive drinking relapse, such as anxiety, dysphoria, irritability, and insomnia (see Figure 1). To this end, medication development for AUD can benefit from the use of a framework for stages of the AUD cycle that is linked to neurocircuitry and that includes protracted withdrawal/negative affect.^56^ Indeed, dysregulation in the brain reward and stress systems that results in the symptoms associated with the protracted withdrawal/negative affect and preoccupation/anticipation stages of the AUD cycle is a neglected focus for AUD drug development. Both repurposed drugs (e.g., gab apentin and mifepristone, a glucocorticoid receptor antagonist)^57^ and new molecular entities (e.g., a vasopressin V1b receptor antagonist)^58^ are all selective for restoring homeostasis in brain stress systems that drive symptoms of protracted withdrawal, and they show promise as emerging new treatments for AUD.

Medications can help restore normal brain functioning, reduce relapse risk, and decrease symptoms of protracted withdrawal (e.g., craving, mood, sleep disturbance), thereby facilitating better engagement in behavioral treatment. Behavioral therapies, in turn, enhance pharmacotherapy response by modifying attitudes and behaviors related to alcohol, increasing healthy life skills, and helping people to stay engaged in recovery.

The Alcohol Treatment Navigator website ( https://alcoholtreatment.niaaa.nih.gov ) was created by the National Institute on Alcohol Abuse and Alcoholism to assist individuals in locating clinicians who provide evidence-based behavioral and/or pharmacological treatments for AUD. Combining evidence-based pharmacological and behavioral treatments for AUD may increase the likelihood of individuals with AUD meeting their goals for recovery.

## Figures and Tables

**Table 1:** Summary of Treatment Parameters for Medications Approved by the FDA for Alcohol Use Disorder

Parameter	Disulfiram* *(oral)*	Naltrexone* *(oral)*	Naltrexone* *(injectable)*	Acamprosate* *(oral)*
**Primary evidence-based outcome**	No drinking Double-blind trials, n.s.^25^ Open-label trials, moderate effect size^25^ Supervised administration trials, large effect size^25^	No heavy drinking NNT = 12^14^ NNT = 8.6^15^	Heavy drinking days WMD = −4.6%^14^	No drinking NNT = 12^14^ NNT = 7.5^15^
**Median trial duration**	6.5 months^25^	3 months^14^	6 months^20^	6 months^14^
**Dosing**	500 mg daily, Weeks 1–2; 250 mg daily thereafter	One 50 mg tablet, daily	One 380 mg injection, monthly	Two 333 mg tablets, 3× daily
**Cost per month** †	$48	$33	$1,308	$142
**Abstinent baseline**	≥ 12 hours (mandatory)‡	≈ 4 days^15^	7 days^20^,‡	≈ 6 days^15^
**Medical contraindications** ‡	Use of metronidazole, paraldehyde, alcohol-containing preparations Severe myocardial disease or coronary occlusion Psychosis	Opioid dependence, withdrawal, or use Acute hepatitis or liver failure	Opioid dependence, withdrawal, or use within 7–10 days Acute hepatitis or liver failure	Severe renal impairment (creatinine clearance *≤* 30mL/min)
**Adverse events**	Neuritis, neuropathy‡ Hepatitis, hepatic failure‡ Psychosis‡ Drowsiness, fatigue‡ Impotence‡ Headache‡ Acne, allergic dermatitis‡ Metallic, garlic aftertaste‡	Dizziness NNH = 16^14^ Nausea NNH = 9^14^ Vomiting NNH = 24^14^	≥ 5% and 2× placebo‡ Vomiting, nausea Injection site reactions Muscle cramps Dizziness, syncope Somnolence, sedation Decreased appetite	Diarrhea 17% (placebo 10%)‡

* Review each drug’s package insert for full prescribing information.

† Monthly cost estimates provided by local discount pharmacy (Costco) and are based on generic formulations when available.

‡ Information derived from package inserts.

*Note:* FDA, U.S. Food and Drug Administration; NNH, a statistical estimate of the number needed to harm for the specified adverse event to occur in one individual; NNT, a statistical estimate of the number needed to treat to achieve the specified outcome in one individual; n.s., not significantly different than placebo; WMD, weighted mean difference.
